# *Hgs* Deficiency Caused Restrictive Cardiomyopathy via Disrupting Proteostasis

**DOI:** 10.7150/ijbs.69024

**Published:** 2022-02-28

**Authors:** Zhenhua Li, Tianle Wang, Chong Xin, Yao Song, Jingyi Kong, Jingping Xu, Qiqi Liu, Yan Teng, Ning Hou, Xuan Cheng, Guan Yang, Wenjia Liu, Bin Zhou, Youyi Zhang, Xiao Yang, Jian Wang

**Affiliations:** 1 State Key Laboratory of Proteomics, Beijing Proteome Research Center, National Center for Protein Sciences, Beijing Institute of Lifeomics, Beijing 100071, China.; 2 Institute of Vascular Medicine, Peking University Third Hospital and Key Laboratory of Molecular Cardiovascular Sciences, Ministry of Education, Key Laboratory of Cardiovascular Molecular Biology and Regulatory Peptides, Ministry of Health, Beijing 100191, China.; 3 The State Key Laboratory of Cell Biology, CAS Center for Excellence in Molecular Cell Science, Shanghai Institute of Biochemistry and Cell Biology, Chinese Academy of Sciences, University of Chinese Academy of Sciences, Shanghai, 200031, China.

## Abstract

The molecular mechanisms underlying restrictive cardiomyopathy (RCM) are not fully understood. Hepatocyte growth factor-regulated tyrosine kinase substrate (HGS) is a vital element of Endosomal sorting required for transport (ESCRT), which mediates protein sorting for degradation and is crucial for protein homeostasis (proteostasis) maintenance. However, the physiological function and underlying mechanisms of HGS in RCM are unexplored. We hypothesized that HGS may play vital roles in cardiac homeostasis. Cardiomyocyte-specific *Hgs* gene knockout mice were generated and developed a phenotype similar to human RCM. Proteomic analysis revealed that *Hgs* deficiency impaired lysosomal homeostasis in cardiomyocytes. Loss of *Hgs* disrupted cholesterol transport and lysosomal integrity, resulting in lysosomal storage disorder (LSD) with aberrant autophagosome accumulation and protein aggregation. Suppression of protein aggregation by doxycycline treatment attenuated cardiac fibrosis, and diastolic dysfunction in *Hgs*-knockout mice. These findings uncovered a novel physiological role of HGS in regulating cardiac lysosomal homeostasis and proteostasis, suggesting that the deficient HGS contributes to LSD-associated RCM-like cardiomyopathy.

## Introduction

Restrictive cardiomyopathy (RCM) is a rare form of myocardial disease, characterized by increased myocardial stiffness, which leads to impaired ventricular filling and diastolic dysfunction 1. RCMs are of heterogenous origins and symptoms, and can be normally classified as infiltrative, non-infiltrative, endomyocardial disorders, and storage diseases 2, 3. Despite its low incidence, RCM is characterized by a poor prognosis and high mortality rate 3. Nevertheless, the mechanisms of idiopathic RCM remain largely unclear.

Compared with most other cells, the survival and function of long-lived post-mitotic cardiomyocytes are more reliant on protein homeostasis (proteostasis). Loss of proteostasis causes the aggregation of aberrant proteins, which in turn causes proteotoxicity and eventually leads to cell death and pathological changes 4. An *in silico* network analysis identified a set of proteotoxicity-associated proteins, whose dysregulation was implicated in myofibrillar myopathies 5. Lysosome is one of the major components of protein quality control (PQC) system that is crucial for the exquisite regulation of cellular proteostasis. Structural abnormality or functional impairment of the lysosomes impairs PQC system, causes proteotoxicity and eventually leads to cell death 4. Lysosomal dysfunction is the cause of lysosomal storage disorders (LSDs) characterized by the accumulation of damaged organelles and proteins in the cell 6. Lysosomal dysfunction has been shown to have significant cardiac manifestations and can induce hypertrophic cardiomyopathy and heart failure 7. However, whether lysosomal dysfunction can cause RCM is rarely known. In addition, although several case reports have revealed the clinical association of LSD and RCM 8, 9. LSD mouse model with RCM symptoms has not been reported.

Endosomal sorting complex required for transport (ESCRT) is a cytosolic protein complex involved in endosomal sorting of ubiquitinated receptors, multivesicular endosome biogenesis, and autophagy 10. The series of four complexes (ESCRT-0, -I, -II, -III)-mediated protein sorting to the lysosome for degradation is an important part of the PQC system. ESCRT-I, -II and ESCRT-III can also be recruited to the damage site and promote sealing of damaged lysosome, suggesting that ESCRTs directly participate in the lysosomal homeostasis 11, 12. Hepatocyte growth factor-regulated tyrosine kinase substrate (HGS) is a major component of the ESCRT-0 complex, which functions in initiating the ESCRT pathway through recognizing ubiquitylated cargo and recruiting the other ESCRT components 10. HGS has been revealed to function in tumor metastasis 13, 14, neurodegeneration 15, 16, as well as the esophageal motility maintenance 17. HGS has a distinct function in transporting low-density lipoprotein-derived cholesterol from the endosomes to the endoplasmic reticulum 18, a process that is tightly associated with lysosomal functions 19, suggesting that HGS might play a role in the maintenance of lysosomal homeostasis. Nevertheless, the physiological function of HGS in cardiac lysosomal homeostasis and proteostasis has not yet been defined.

In the present study, we find that HGS is indispensable for cardiac proteostasis through modulating cholesterol transport and lysosomal integrity. Loss of *Hgs* in cardiomyocytes results in RCM characterized by normal left ventricular (LV) chamber size and wall thickness with dilated atria, increased ventricular stiffness and diastolic dysfunction. Importantly, inhibition of protein aggregation by doxycycline (Dox) treatment attenuates the RCM of *Hgs* knockout mice. Our findings demonstrate a causal link between HGS deficiency and LSD-associated RCM, suggesting a novel therapeutic strategy for treating RCM.

## Materials and Methods

### Animals

To obtain mice with targeted deletion of *Hgs* in the heart, homozygous mice for the floxed *Hgs* 17 allele were bred with α-MHC-Cre transgenic mice 20, in which the expression of Cre recombinase is driven by the α-MHC promoter. Mice were kept on a mix C57BL/6J X 129/SV background. To genotype the mice, genomic DNA was isolated from mouse tail biopsies and analyzed by polymerase chain reaction (PCR) using the following specific primers: *Hgs*, 5′-CCTGGTGTCCTTGGATCTCCT-3′ and 5′-GAGCCACTCTTGTAGCCTTGC-3′; *Cre*, 5′-GCCTGCATTACCGGTCGATGC-3′ and 5′-CAGGGTGTTATAAGCAA TCCC-3′.

Temporally controlled cardiomyocyte-specific *Hgs*-knockout mice were generated by breeding mice bearing *Hgs* floxed alleles with transgenic a-MHC-MerCreMer mice 21. We administered an intraperitoneal injection of 100 mg/kg (body weight) of tamoxifen (Sigma, St. Louis, MO, USA) or vehicle to 4-week-old male mice for 4 consecutive days. At 1.5 months after the final injection, echocardiograph analysis was performed, and heart tissues were harvested for analysis.

All experiments were performed blinded to genotypes.

### Isolation of adult cardiomyocytes and non-cardiomyocytes

After treated with 200 μL heparin (3.5 mg/mL) for 15 minutes, the mice were anesthetized intraperitoneally with Avertin (250 mg/kg). A few minutes later, the heart was harvested and put into the cold oxygenated calcium-free solution. After cutting off all blood vessels, connective tissue and the above aortic arch outside the aorta, the heart was ligated to the perfusion fluid-filled aortic cannula and perfused with calcium-free solution (137 mM NaCl, 5.4 mM KCl, 1.2 M NaH_2_PO_4_·2H_2_O, 1.2 mM MgCl_2_·6H_2_O, 20 mM HEPES, 10 mM taurine, and 10 mM Glucose·H_2_O, pH 7.35) at a rate of 3 mL/min for about 10 times. Next, the heart was perfused with an enzyme buffer (0.714 mg/mL type II collagenase, 1 mg/mL taurine and 50 μM CaCl_2_) for about 10 - 20 minutes at the same flow rate at 37 °C. Upon softening of the heart tissue, the heart was removed from the cannula, and the left ventricle was separated and minced. The digested tissue was rigorously pipetted up and down for several times, and dissociated cells were centrifuged for 3 minutes at 100×g to pellet cardiomyocytes. The supernatant was transferred to a new 15 mL centrifuge tube, and centrifuged for 3 minutes at 300×g to collect non-cardiomyocytes.

### Morphological and histological analysis

For pathological studies, hearts of mice were isolated, fixed overnight in 4% paraformaldehyde, embedded in paraffin, and serially sectioned every 5 μm for gross and histological examination. The sections were routinely stained using hematoxylin and eosin to examine the myocardium, Masson's trichrome staining for assessment of fibrosis, wheat germ agglutinin (WGA) staining to assess cardiomyocyte cross-sectional area, and von Kossa staining for detection of calcium. For Masson's trichrome staining, 10-12 pictures at 4X magnification were obtained for each sample, and were stitched together in Photoshop CC with Photomerge function. The blue colour was defined as fibrosis area, which was drawn manually and analysed by Image-Pro Plus. The quantification result was a measurement of percentage of the fibrosis area in the cross-section of whole heart.

### Immunofluorescence

Briefly, sections were incubated with anti-cleaved-LC3B (cat. no. AP1806a; Abgent, San Diego, CA, USA), anti-LAMP1 (cat. no. ab25245; Abcam, Cambridge, UK), anti-CD63 (cat. no. ab217345; Abcam), anti-LGALS3 (cat. no. sc-23938; Santa Cruz Biotechnology, Dallas, TX, USA), anti-ubiquitin (cat. no. 3936; Cell Signaling Technology), anti-BAG3 (cat. no. 10599-1-AP; Proteintech, Rosemont, IL, USA), anti-CRYAB (cat. no. ADI-SPA-222; Enzo Life Sciences, Farmingdale, NY, USA), and anti-FLNC (cat. no. ab180941; Abcam) primary antibodies at 4°C overnight after antigen retrieval and horseradish peroxidase (HRP) inactivation. HRP-conjugated secondary antibodies (ZSGB-BIO, Beijing, China) were then applied. Immunopositive cells were visualized with TSA Plus Cyanine3 Working Solution (PerkinElmer, Waltham, MA, USA). The antibodies were then removed by incubating the slides in citrate buffer for 7 min at 100 °C. In addition, sections were costained with anti-TNNT2 (cat. no. BS6013; Bioworld Technology, St. Louis Park, MN, USA) or anti-α-laminin (cat. no. L9393; Sigma-Aldrich) primary antibodies and HRP-conjugated secondary antibodies. Immunopositive cells were visualized with TSA Plus Fluorescein Working Solution (PerkinElmer). The sections were then stained with Hoechst and observed.

### Echocardiography analysis

Echocardiography was performed in *Hgs*-cKO mice and sex-matched littermate controls, using a Vevo 2100 system (VisualSonics, Toronto, Canada) equipped with a 30 MHz transducer. Anesthesia was induced with 3% isoflurane and maintained with 1.5% isoflurane during constant monitoring of temperature, respiration rate, and electrocardiogram. Left ventricle (LV) wall thickness and chamber dimensions were obtained from M-mode images at the mid-papillary level in the parasternal short axis view, and the ejection fraction (EF) and fractional shortening (FS) were calculated.

Pulse-wave Doppler and tissue Doppler imaging were recorded from the apical 4-chamber view. The peak early transmitral flow velocity (E), peak late transmitral flow velocity (A), E-wave deceleration time (EDT), ratio of E to A (E/A), peak early diastolic mitral annular velocity (E'), peak late diastolic mitral annular velocity (A'), and ratio of E' to A' (E'/A') were measured and analyzed.

To measure LAID, we obtained a parasternal long axis view of the heart in B-mode, then adjusted the scanhead to clearly show the left ventricle, aortic root, mitral valve leaflets and left atrium. The internal diameter of left atrium was measured by placing calipers from lower edge of the aortic root to posterior border of the left atrium as shown in the following illustration.

### Western blotting

Western blotting was carried out on myocardial extracts as described previously 20, 22. Western blotting was performed from whole heart lysate homogenized in RIPA lysis buffer (C1053, Applygen) with protease inhibitors (04693159001, Roche). Proteins were quantified using Pierce BCA Protein Assay Reagent (23225, Thermo Fisher). Thirty micrograms of proteins were electrophoretically separated on an 8%, 10% or 12% SDS-polyacrylamide gel when appropriate and transferred onto a PVDF membrane. The membrane was blocked for 1 hour in 5% milk in PBST, and incubated with primary antibodies at 4 °C overnight. The following antibodies were used: anti-HGS (cat. no. ALX-804-382; Enzo Life Sciences), anti-SQSTM1 (cat. no. 5114; Cell Signaling Technology), anti-LC3B (cat. no. L7543; Sigma-Aldrich), anti-LAMP1 (cat. no. ab25245; Abcam), anti-CRYAB (cat. no. ADI-SPA-222; Enzo Life Sciences), anti-BAG3 (cat. no. 10599-1-AP; Proteintech), anti-DES (cat. no. 5332; Cell Signaling Technology), anti-ANKRD1 (cat. no. sc-365056; Santa Cruz Biotechnology, Dallas, TX, USA), anti-CD63 (cat. no. ab217345; Abcam), anti-LGALS3 (cat. no. 14979-1-AP; Proteintech), anti-CTSD (cat. no. 21327-1-AP; Proteintech), anti-CTSB (cat. no. ab214428; Abcam), anti-ANP (cat. no. ab225844; Abcam), anti-Myh7 (cat. no.22280-1-AP; proteintech) and anti-GAPDH (cat. no. TA-08; ZSGB-BIO). Then, the membrane was washed and incubated with Peroxidase-Conjugated Goat anti-Rabbit IgG (ZB-2301, ZSGB-BIO) or Peroxidase-Conjugated Goat anti-Mouse IgG (ZB-2305, ZSGB-BIO). Finally, the immunoblot signal was detected using Enlight Western blotting detection reagents (29100, Engreen Biosystem). The intensities of the bands were quantified by densitometry using the ImageJ software.

### Real-time quantitative PCR

Total RNA was isolated from heart tissues and neonatal cardiomyocytes using TRIzol (Thermo Fischer Scientific, Waltham, MA, USA). cDNA was synthesized using a SuperRT One Step RT-PCR Kit (CWBIO, Bejing, China) and subjected to real-time PCR using SYBR Green Real-time PCR Master Mix (TOYOBO, Osaka, Japan) on a 7500 Fast Real-Time PCR System (Applied Biosystems, Foster City, CA, USA).

GAPDH was used as the reference gene. The following primers were used: GAPDH, 5′-TGCCCAGAACATCATCCCT-3′ and 5′-GGTCCTCAGTGTAGCCCAA G-3′; *Myh7*, 5′-GTGAAGGGCATGAGGAAGAGT-3′ and 5′-AGGCCTTCACCTTC AGCTGC-3′; *Nppa*, 5′-GCCGGTAGAAGATGAGGTCA-3′ and 5′-GGGCTCCAAT CCTGTCAATC-3′; *Nppb*, 5′-GCTCTTGAAGGACCAAGGCCTCAC-3′ and 5′-GATCCGATCCGGTCTATCTTGTGC-3′; *Col1a1*, 5′-AGCGAAGAACTCATACA GCCG-3′ and 5′-TTGGAGCAGCCATCGACTAG-3′; and *Col3a1*, 5′-GCCTCCCAGAACATTACATACC-3′ and 5′-GGGTAGTCTCATTGCCTTGC-3′.

### Assessing autophagic flux in the heart

Chloroquine was dissolved in saline and intraperitoneally injected in adult (2-month-old) *Hgs*^fl/fl^ and* Hgs*-cKO mice at a dosage of 60 mg/kg. Mice were maintained in absence of food. Four hours after injection, mice were sacrificed, and hearts were used to perform molecular and biochemical analyses. A group of *Hgs*^fl/fl^ mice injected with the same amount of the vehicle was also tested.

### Filipin staining

Frozen heart sections were incubated with filipin (cat. no. SEA0088, Sigma-Aldrich) at a working concentration of 0.5 mg/mL dilated in phosphate-buffered saline (PBS) for 30 minutes in the dark at room temperature. Sections were mounted after washing in PBS. Images were visualized Imager A2 microscope (Carl Zeiss AG) using an ultraviolet filter set and captured by the SPOT RT3 microscope camera (Micro Video Instruments, Inc). ImageJ software was used to determine the fluorescent density of each image.

### Mass spectrometry (MS)-based proteomic analysis

Proteomic analysis was performed in 20-day-old *Hgs*^fl/fl^ and *Hgs*-cKO hearts. Proteins from heart tissues were extracted and transferred into 10-kDa filter units, washed twice with 8 M urea and 50 mM ammonium bicarbonate, and digested using trypsin at an enzyme-to-protein mass ratio of 1:50 overnight at 37 °C. The reaction was stopped by addition of 1% formic acid.

Samples were analyzed on a Q Exactive HF mass spectrometer (Thermo Fisher Scientific) connected to an Easy-nLC 1000 liquid chromatography system (Thermo Fisher Scientific). Dried peptide samples were redissolved in 0.1% formic acid in water and loaded onto a trap column (100 μm × 20 mm; particle size, 3 μm; pore size, 120 Å; SunChrom, Friedrichsdiorf, Germany) with a maximum pressure of 280 bar and then separated on a homemade 150 μm × 30 cm silica microcolumn (particle size, 1.9 μm; pore size, 120 Å; Dr. Maisch GmbH, Ammerbuch, Germany) with a gradient of 5-35% mobile phase (acetonitrile and 0.1% formic acid) at a flow rate of 600 nL/min for 150 min. MS analysis was performed with one full scan (300-1400 m/z, R = 120,000 at 200 m/z) at an automatic gain control target of 3e6 ions, followed by up to 30 data-dependent MS/MS scans with higher-energy collision dissociation (target 2e4 ions; max injection time, 40 ms; isolation window, 1.6 m/z; normalized collision energy, 27%), detected by Orbitrap analysis (R = 15,000 at 200 m/z). The dynamic exclusion of previously acquired precursor ions was enabled at 18 s.

Raw MS files were managed by MaxQuant software (version 1.6.0.16). MS/MS-based peptide identification was carried out with the Andromeda search engine in MaxQuant, which uses a target-decoy approach to identify peptides and proteins at a false-discovery rate of less than 1%. The mouse protein database from NCBI was used as the forward database. The reverse database for the decoy search was generated automatically in MaxQuant. Enzyme specificity was set to “trypsin,” and a minimum number of seven amino acids was required for peptide identification. Default settings were used for the variable (acetylation [protein N-terminus] and oxidation [methionine]) and for fixed modifications (carbamidomethylation).

Proteins showing a fold increase of at least 2.0 or a fold decrease of at least 0.5, with p values of less than 0.05, were considered differentially expressed. Deregulated proteins due to the absence of *Hgs* were analyzed by Gene Set Enrichment Analysis (GSEA) with Kyoto Encyclopedia of Genes and Genomes (KEGG) modules.

### Administration of doxycycline (Dox)

8-week-old* Hgs*-cKO mice (4 females and 4 males) and *Hgs*^fl/fl^ mice (3 females and 4 males) were randomly divided into four groups to be treated with or without Dox for 8 weeks. Dox (6 mg/mL; Sigma-Aldrich Corp) was given in drinking water containing 5% sucrose. The control group was given drinking water containing 5% sucrose without Dox.

### Statistics

All data are expressed as means ± standard errors of the means (SEM). Comparisons between experimental groups were performed using two-tailed Student's *t-*tests. A Two-Way ANOVA with multiple comparison analysis was used in the Dox treatment study. Results with *p* values of less than 0.05 were considered statistically significant.

### Study approval

All animal procedures were undertaken in accordance with the protocols approved by the Animal Experiment Committee of the Beijing Institute of Lifeomics and conformed to the US National Institutes of Health Guide for the Care and Use of Laboratory Animals.

## Results

### Deletion of Hgs in cardiomyocytes resulted in RCM-like cardiomyopathy

**Figure 1:**
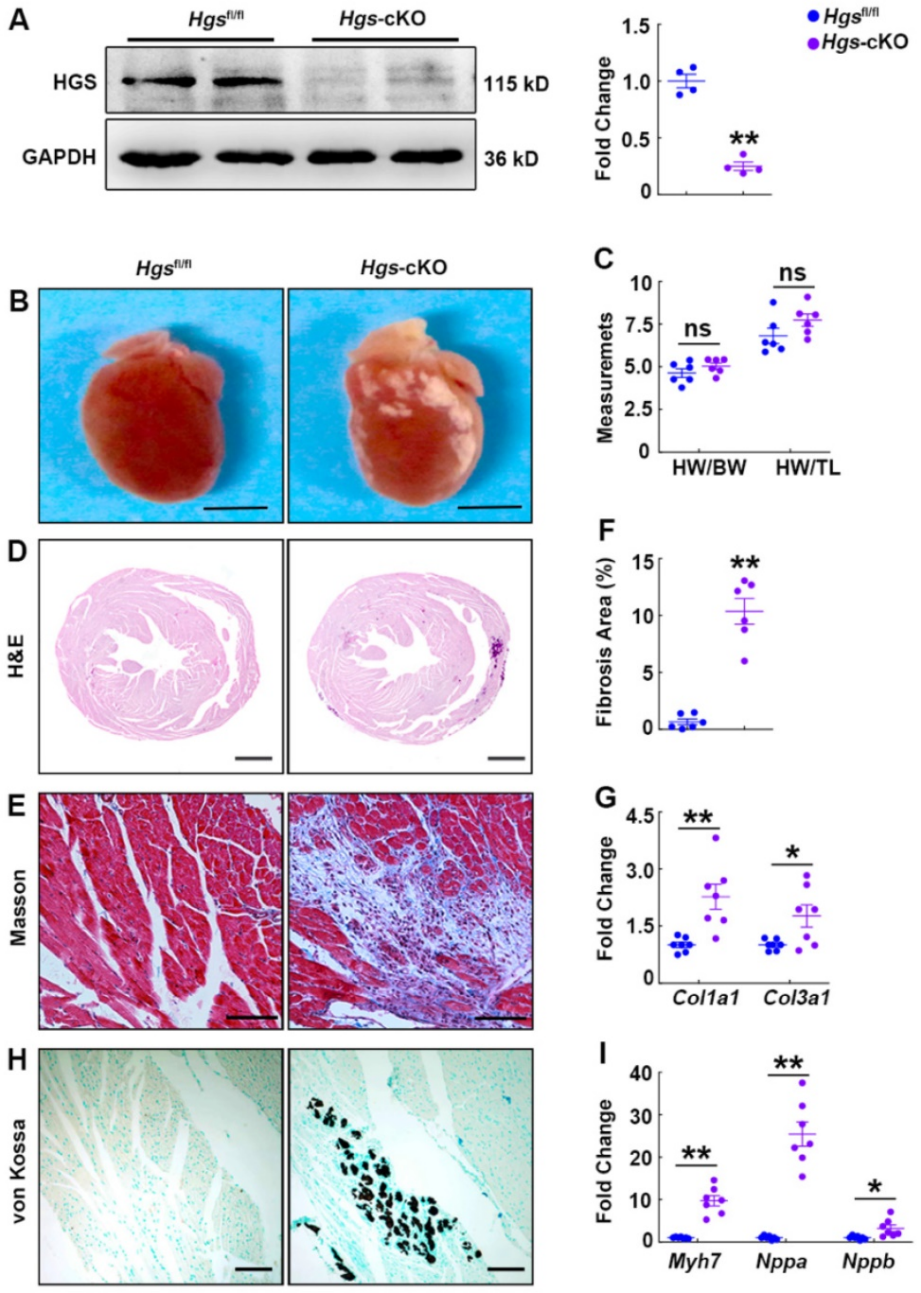
**Deletion of *Hgs* in cardiomyocytes resulted in cardiac fibrosis, calcification, and upregulation of fetal genes.** (**A**) Western blotting of HGS expression in *Hgs*-cKO and *Hgs*^fl/fl^ hearts. Quantification is shown on the right. **p < 0.01 (means ± SEM, n = 4). (**B**) Gross morphology of *Hgs*^fl/fl^ and *Hgs*-cKO hearts at 3 months. Scale bars, 3 mm. (**C**) Ratios of heart weight to body weight or tibia length in 3-month-old *Hgs*-cKO and *Hgs*^fl/fl^ mice (means ± SEM, n = 6). (**D**) Hematoxylin and eosin (H&E) staining of transverse sections from 3-month-old *Hgs*^fl/fl^ and *Hgs*-cKO hearts. Scale bars, 1 mm. (**E**) Masson's trichrome staining of sections from 3-month-old *Hgs*^fl/fl^ and *Hgs*-cKO hearts. Scale bars, 100 µm. (**F**) Quantification of fibrotic areas in *Hgs*-cKO hearts. **p < 0.01 (means ± SEM, n = 6). (**G**) Real-time PCR analyses of *Col1a1* and *Col3a1* mRNA levels in 3-month-old *Hgs*^fl/fl^ and *Hgs*-cKO mice. *p < 0.05, **p < 0.01 (means ± SEM, n = 7). (**H**) Heart sections were stained with von Kossa stain. Scale bars, 50 µm. (**I**) Real-time PCR analyses of *Myh7*, *Nppa,* and *Nppb* mRNA levels in 3-month-old *Hgs*^fl/fl^ and *Hgs*-cKO mice. *p < 0.05, **p < 0.01 (means ± SEM, n = 7).

To determine the physiological function of HGS in the maintenance of cardiac homeostasis, we bred cardiomyocyte-specific α*-*MHC*-Cre* transgenic mice 20 with *Hgs* floxed mice 17 to knockout *Hgs* gene specifically in cardiomyocytes. Immunoblot analysis of heart extracts and real-time PCR analysis of adult cardiomyocytes confirmed the loss of HGS in cardiomyocytes (Figure 1A, Supplemental Figure 1, A-B). The α-MHC*-Cre;Hgs*^fl/fl^ (hereafter *Hgs*-cKO) mice were born normally (Supplemental Table 1) and had normal cardiac structure. There was no premature death, and the mutant mice were fertile. We observed 20 mice of each genotype for about 1 year, and failed to find any significant difference in the survival rate between littermate control *Hgs*^fl/fl^ and *Hgs*-cKO mice (Supplemental Table 1). Body weight and cardiac size were unchanged in *Hgs*-cKO mice compared with that in *Hgs*^fl/fl^ mice (Figure 1, B-D and Supplemental Figure 1, C-E). WGA staining of hearts also revealed that the cross-section area (CSA) of cardiomyocytes in *Hgs*-cKO mice was comparable to that in *Hgs*^fl/fl^ mice (Supplemental Figure 1F). However, the hearts of *Hgs*-cKO mice appeared pale, with abnormal white patches on the surface (Figure 1B). As revealed by Masson's trichrome staining, dense fibrosis with collagen bundle formation appeared in *Hgs*-cKO hearts (Figure 1E and Supplemental Figure 1G). In addition, quantification of the fibrotic areas and detection of the profibrotic genes type I collagen alpha 1 (*Col1a1*) and type III collagen alpha 1 (*Col3a1*) further verified the enhanced cardiac fibrosis in *Hgs*-cKO hearts (Figure 1, F-G). Multiple calcific deposits in the cardium of *Hgs*-cKO mice were revealed by von Kossa staining (Figure 1H). Reactivation of the fetal genes encoding β-myosin heavy chain (*Myh7*), atrial natriuretic peptide (*Nppa*), and brain natriuretic peptide (*Nppb*) was evident in the heart tissues of *Hgs*-cKO mice (Figure 1I and Supplemental Figure 1H).

**Figure 2:**
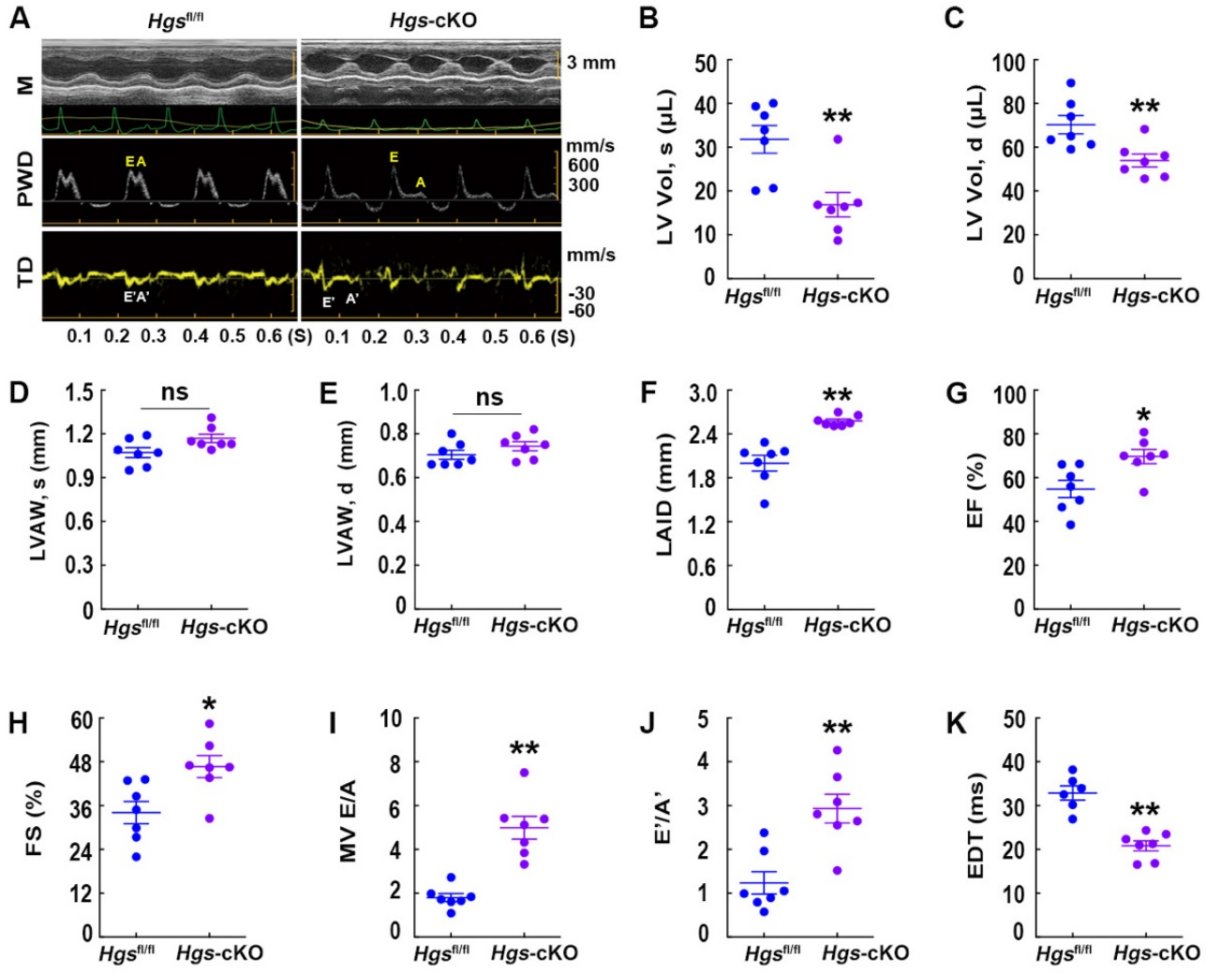
***Hgs*-cKO mice developed cardiac diastolic dysfunction.** (**A**) Representative echocardiographic images of M-mode (M), pulsed-wave Doppler (PWD), and tissue Doppler (TD) echocardiograms of 3-month-old *Hgs*^fl/fl^ and *Hgs*-cKO mice. (**B, C**) Measurements of the LV volume in diastole (LV Vol, d) and systole (LV Vol, s). (**D, E**) Measurements of the LV anterior wall thickness in systole (LVAW, s) and diastole (LVAW, d). (**F**) Measurements of the left atrium internal diameter (LAID). (**G, H**) Quantification of ejection fraction (EF) and fractional shortening (FS).** (I, J)** Quantification of the mitral valve E/A and E'/A'. (**K**) Measurements of E-wave deceleration time (EDT). *p < 0.05, **p < 0.01 (means ± SEM, n = 7).

M-mode echocardiography demonstrated that *Hgs*-cKO hearts had normal left ventricle (LV) wall thickness, decreased LV volume and internal diameters, and increased left atrium internal diameter (Figure 2A-F, Supplemental Figure 1I-L and Supplemental Table 2). Indices of systolic function, ejection fraction (EF) and fractional shortening (FS), were slightly enhanced in *Hgs*-cKO mice (Figure 2G-H), indicating a preserved systolic function. Remarkably, echo-Doppler measurements in *Hgs*-cKO mice showed abnormal LV relaxation and elevated filling pressure, as demonstrated by the significantly increased E/A ratio (≥ 2) and decreased EDT (Figure 2, A and I-K and Supplemental Table 3).

We also performed histological, molecular and echocardiographic analysis in 3-month-old α-MHC*-Cre* transgenic mice, which showed no obvious difference from wild type mice (Supplemental Figure 2), ruling out the possibility that RCM is caused by α-MHC*-Cre* transgene.

Our results indicated that cardiomyocyte-specific *Hgs*-knockout mice developed cardiomyopathy with interstitial fibrosis, enlarged atrial volume, and diastolic dysfunction, all of which are common features of RCM 4.

### Hgs deficiency impaired the lysosomal homeostasis of cardiomyocytes

**Figure 3:**
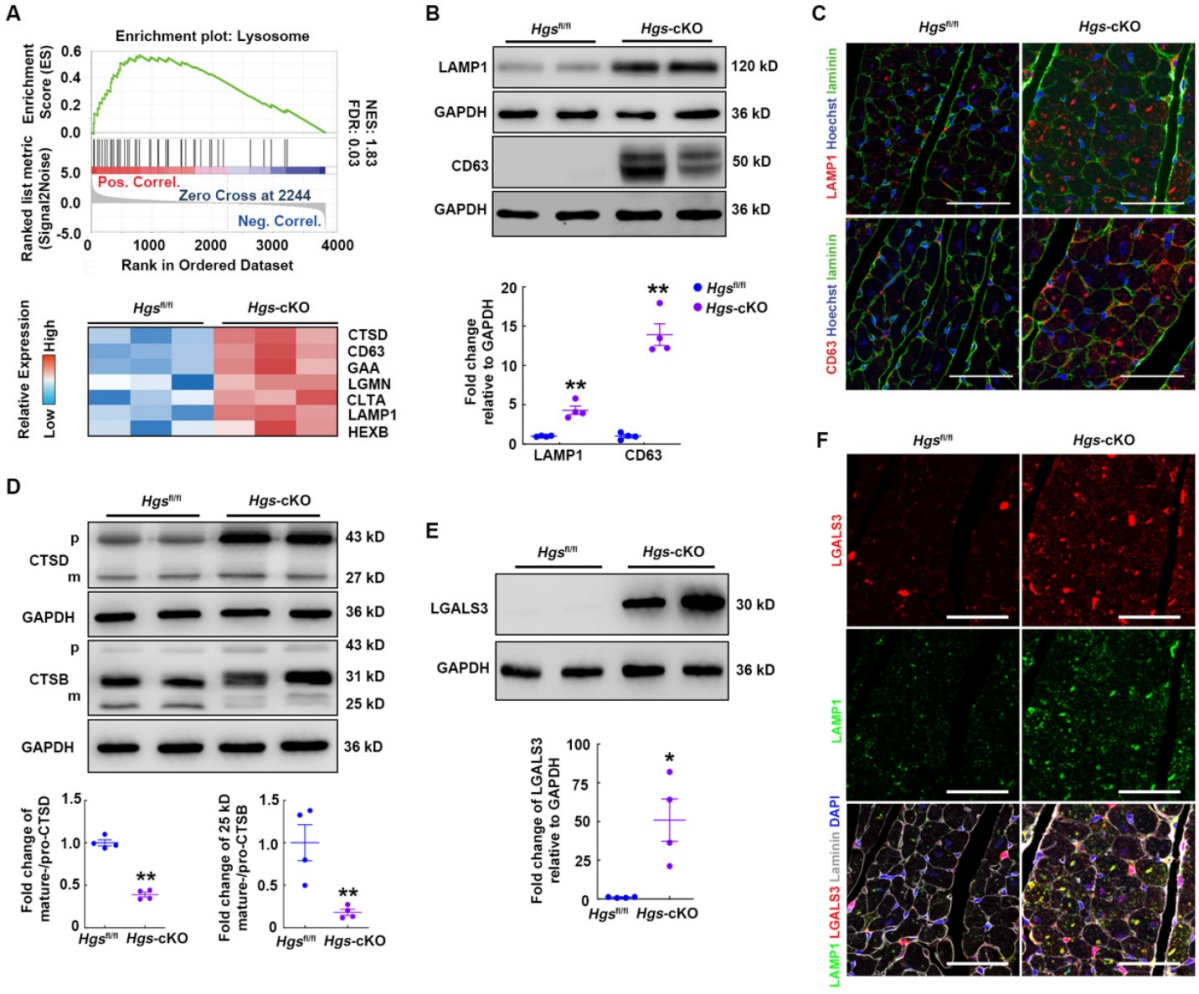
***Hgs* ablation impaired lysosome homeostasis.** (**A**) Enrichment plot showing the lysosome pathway enriched in *Hgs*-cKO mice. The heatmap of genes encoding key proteins in the lysosome pathway is shown in the lower panel. (**B**) Western blotting of the lysosomal marker LAMP1 and CD63 in ventricular extracts from 3-month-old *Hgs*-cKO and *Hgs*^fl/fl^ hearts. Quantification is shown below. **p < 0.01 (means ± SEM, n = 4). (**C**) Representative immunofluorescence images of ventricular sections from 3-month-old *Hgs*^fl/fl^ and *Hgs*-cKO mice costained for laminin (green) and LAMP1 or CD63 (red). Scale bars, 50 µm. (**D**) Western blotting of CTSD and CTSB in ventricular extracts from 3-month-old *Hgs*-cKO and *Hgs*^fl/fl^ hearts. Quantification is shown on the right. **p < 0.01 (means ± SEM, n = 4). (**E**) Western blotting of LGALS3 in ventricular extracts from 3-month-old *Hgs*-cKO and *Hgs*^fl/fl^ hearts. Quantification is shown on the right. **p < 0.01 (means ± SEM, n = 4). (**F**) Representative confocal fluorescent images of ventricular sections from 3-month-old *Hgs*^fl/fl^ and *Hg*s-cKO mice immunostained for LGALS3 (red) and LAMP1 (green). Scale bars, 50 µm.

To explore the molecular mechanisms through which *Hgs* deficiency caused RCM, we performed quantitative proteomic analysis of heart tissues and screened 202 upregulated and 99 downregulated proteins for further analysis (Supplemental Figure 3A). Gene set enrichment analysis (GSEA) with Kyoto Encyclopedia of Genes and Genomes (KEGG) revealed that pathways regulating the extracellular matrix, lysosome, and proteasome were significantly enriched and upregulated in *Hgs*-cKO mice (Supplemental Figure 3B and Supplemental Table 4). We then focused on the lysosome pathway, which acts as part of the PQC systems and plays important roles in cardiac homeostasis 23. Heatmap of Lysosome pathway components showed that loss of *Hgs* increased the expression of the lysosomal membrane proteins, CD63 and LAMP1 (Figure 3A). Western blot and immunofluorescence analyses confirmed that LAMP1 and CD63 were upregulated, and that the number and size of lysosomes increased in *Hgs*-cKO myocardium (Figure 3B-C), suggesting that *Hgs* deficiency caused accumulated and enlarged lysosomes in cardiomyocytes.

The accumulation of enlarged lysosomes appears in several disorders which is accompanied by deficiency of lysosomal enzymes 24. Lysosomes contain numerous acid hydrolases that involved in the degradation of a broad variety of cargos. Heatmap of lysosome pathway components in Figure 3A showed that several lysosomal acid hydrolases, such as Cathepsin D (CTSD), Cathepsin B (CTSD), alpha glucosidase (GAA), and hexosaminidase B (HEXB), were significantly upregulated in Hgs-cKO hearts. The cathepsins are a major class of lysosomal protease, which are synthesized as immature pro-cathepsins that are proteolytically processed to form mature cathepsins. Western blot showed that pro-CTSD and pro-CTSB were both increased, consistent with the accumulated lysosomes in Hgs-cKO hearts. However, the mature-CTSD and the 25/26 kD mature-CTSB normalized to their immature forms were significantly downregulated (Figure 3D), suggesting the impaired maturation of CTSD and CTSB in *Hgs*-cKO hearts.

The alterations of the cathepsins maturation and the increasement of both size and number of lysosomes reflected lysosome damage. To investigate whether lysosomes were impaired in *Hgs*-cKO cardiomyocytes, we detected the expression of galectin 3 (LGALS3), a marker of lysosome membrane permeabilization (LMP) 25, and found LGALS3 was increased in *Hgs*-cKO hearts (Figure 3E). We also performed immunostaining to further confirm that the number of LGALS3-positive puncta was significantly increased, and that most enlarged LAMP1-positive lysosomes were LGALS3-positive, indicating a higher percentage of damaged lysosomes in *Hgs*-cKO hearts (Figure 3F). These data indicated that deficiency of *Hgs* impaired the lysosomal homeostasis of cardiomyocytes.

### Hgs-cKO hearts resemble LSD with cholesterol accumulation and autophagic impairment

**Figure 4:**
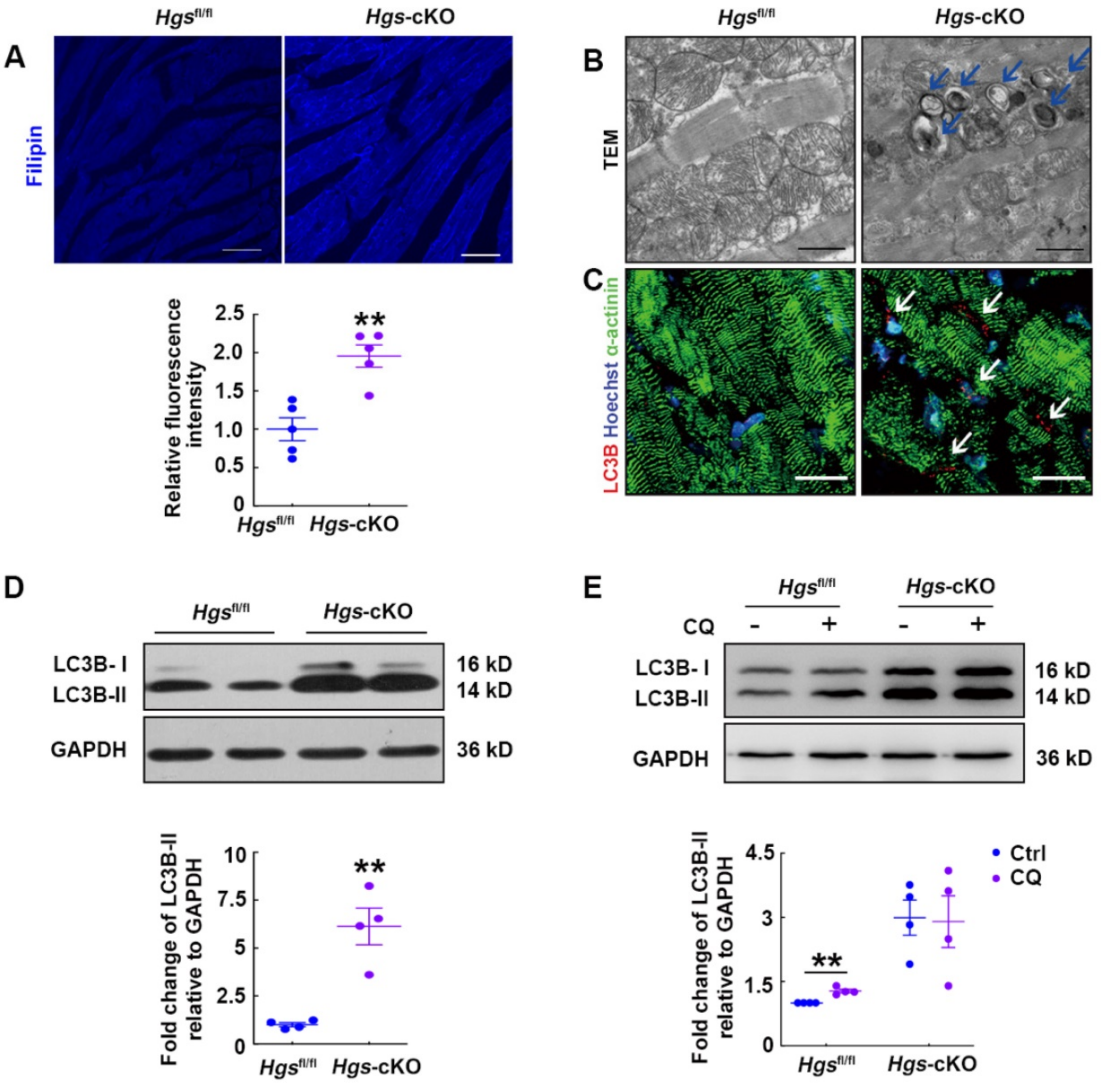
***Hgs*-cKO mice developed LSD with cholesterol accumulation and autophagic impairment.** (**A**) Filipin staining for free cholesterol in 3-month-old *Hgs*-cKO and *Hgs*^fl/fl^ hearts. Quantification of relative fluorescence intensity is shown below. **p < 0.01 (mean ± SEM, n = 5). Scale bars, 100 µm. (**B**) Transmission electron micrographs of ventricular sections from 3-month-old *Hgs*^fl/fl^ and *Hgs*-cKO mice. The arrows indicate autophagosomes. Scale bars, 1 µm. (**C**) Representative confocal fluorescent images of ventricular sections from 3-month-old *Hgs*^fl/fl^ and *Hg*s-cKO mice immunostained for cleaved-LC3B (red) and α-actinin (green). Scale bars, 20 µm. (**D**) Western blotting of LC3B in ventricular extracts from 3-month-old *Hgs*-cKO and *Hgs*^fl/fl^ hearts. Quantification is shown in the lower panel. **p < 0.01 (means ± SEM, n = 4). (**E**) Western blotting on ventricular extracts from 8-week-old *Hgs*^fl/fl^ and *Hg*s-cKO mice treated either with saline (Ctrl) or the chloroquine (CQ). The LC3B-II/GAPDH ratio was evaluated in Ctrl- and CQ-treated *Hgs*^fl/fl^ and *Hgs*-cKO hearts. **p < 0.01 (means ± SEM, n = 4).

The expanded lysosomal compartment is a common feature of most LSDs, and is caused by defects in many different aspects of lysosomal homeostasis, including the exportation of substrates from the lysosome 24. It has been proved that HGS is required for the endo-lysosomal transport of cholesterol to the endoplasmic reticulum 18, so we speculated that *Hgs* deletion might result in cholesterol accumulation. Filipin staining was then performed and verified that *Hgs* deficiency led to accumulation of free intracellular cholesterol in cardiomyocytes (Figure 4A). As cholesterol accumulation has been found to impair lysosomal fusion and the function of soluble N-ethylmaleimide-sensitive factor attachment protein receptors (SNAREs) 26, our data suggested that disruption of lysosomal homeostasis in *Hgs*-cKO mice might be due to cholesterol accumulation.

LSDs are associated with defective autophagy flux in most cases, and autophagosomes are accumulated in LSD due to defects in the lysosomal degradation step. We then used transmission electron microscope to analyze the ultrastructural features of *Hgs*-cKO hearts and confirmed the accumulation of autophagosomes (Figure 4B). Consistent with this, immunofluorescence showed abundant cleaved-LC3B-positive puncta aggregated in the *Hgs*-cKO myocardium (Figure 4C). We also detected increased protein levels of lipidated LC3B (LC3B-II) (Figure 4D). To further confirm that deletion of *Hgs* caused the block of the autophagy flux, we treated adult *Hgs*^fl/fl^ and *Hgs*-cKO mice with chloroquine (CQ), an inhibitor of autophagosome/lysosome fusion. The result showed that CQ increased the LC3B-II in the hearts of *Hgs*^fl/fl^ mice without causing significant changes in the *Hgs*-cKO hearts (Figure 4E), suggesting that *Hgs* loss impaired autophagic flux. Detection of mTOR activity showed that phosphorylation of mTOR at both Ser2481 and Ser2448 sites 27 were unchanged in the *Hgs*-cKO hearts (Supplemental Figure 4A), indicating that accumulation of autophagosomes in *Hgs*-cKO hearts was not due to the upregulation of autophagic induction.

We further infected the neonatal cardiomyocytes with adenovirus encoding monomeric red fluorescent protein (mRFP)-green fluorescent protein (GFP)-LC3B, an autophagy tandem sensor that can detect autophagy flux in real-time 28. RFP and GFP were both expressed in autophagosomes, yielding yellow signals. When lysosomes and autophagic flux were normal, GFP signals were lost upon acidification of the autophagosomes fused with lysosomes. We observed yellow and red puncta in control cells, indicating a basal level of autophagosome and autolysosome formation (Supplemental Figure 4B). Similar to bafilomycin A1 (BFA)-treated cells, *Hgs*-knockout cells exhibited increased double-positive puncta and decreased the number of RFP single-positive puncta (Supplemental Figure 4B), demonstrating that *Hgs* deletion impaired the autophagic flux in cardiomyocytes.

Taken together, these data showed that the histopathological phenotype of *Hgs*-cKO hearts resembled human LSD.

### Proteostasis was impaired in Hgs-cKO cardiomyocytes

**Figure 5:**
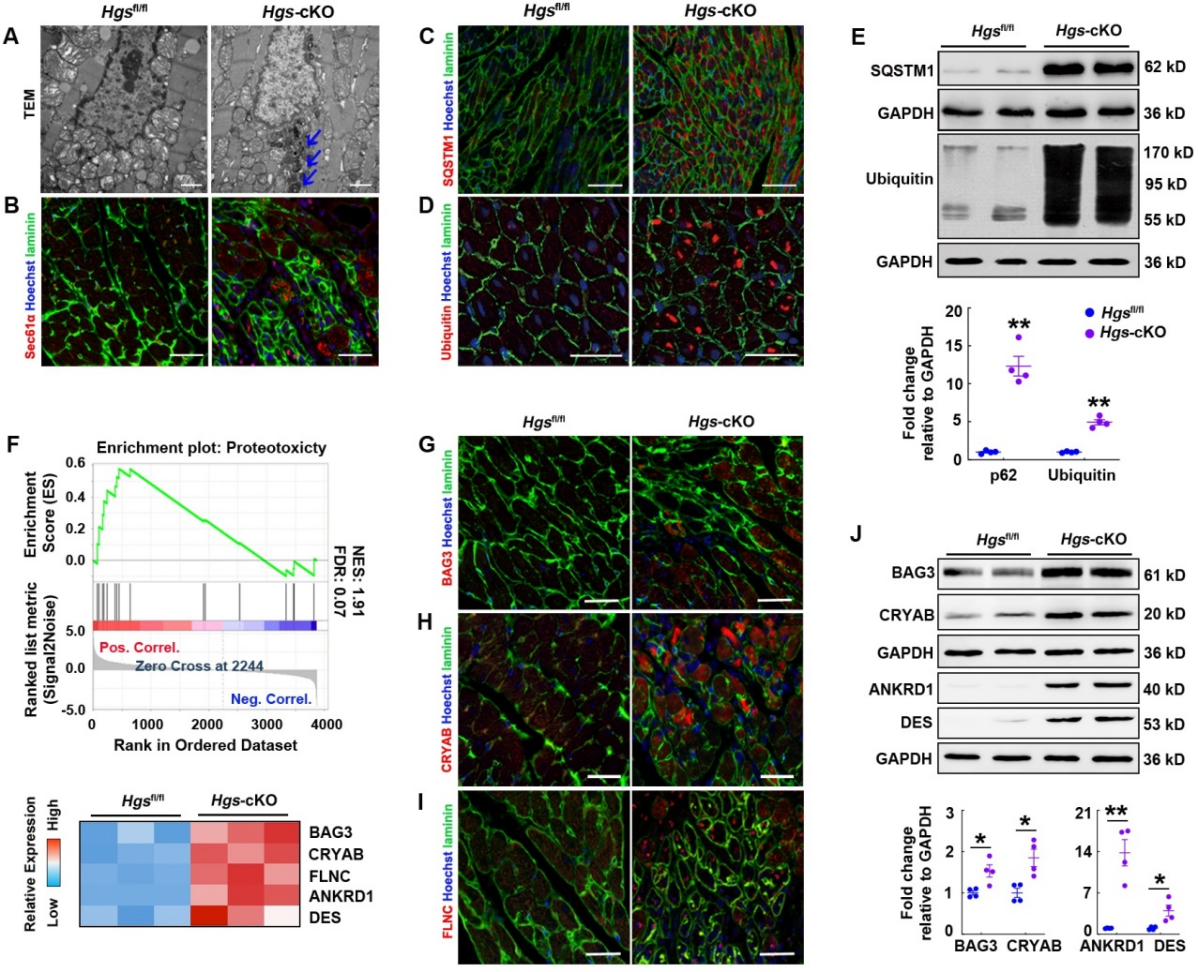
** Proteostasis was impaired in *Hgs*-cKO cardiomyocytes.** (**A**) Transmission electron micrographs of ventricular sections from *Hgs*^fl/fl^ and *Hgs*-cKO mice. The arrows show electron-dense depositions in *Hgs*-cKO cardiomyocytes. Scale bars, 1 µm. (**B**) Representative immunofluorescent images showing increased protein aggregates in *Hgs-*cKO hearts. Staining for Sec61α (red) and laminin (green) was used for detecting aggresomes and cardiomyocytes, respectively. Scale bars, 50 µm. (**C**) Representative immunofluorescent images showing increased SQSTM1 aggregates in *Hgs*-cKO hearts. Scale bars, 100 µm. (**D**) Immunofluorescence analyses of 3-month-old ventricular sections from *Hgs*^fl/fl^ and *Hgs*-cKO mice costained with ubiquitin (red) and laminin (green). Scale bars, 20 µm. (**E**) Western blotting of SQSTM1 and ubiquitinated protein in ventricular extracts from 3-month-old *Hgs*-cKO and *Hgs*^fl/fl^ hearts. Quantifications are shown in the lower panel. **p < 0.01 (means ± SEM, n = 4). (**F**) GSEA of proteomic data showed that the proteins encoded by proteotoxicity-associated genes were enriched and upregulated in 20-day *Hgs*-cKO hearts. The heatmap of genes is shown in the lower panel. (**F-H**) Representative immunofluorescent images of ventricular sections from 2-month-old *Hgs*^fl/fl^ and *Hgs*-cKO mice co-stained with BAG3 (**F**)/ CRYAB (**G**)/ FLNC (**H**) and laminin (green). Scale bars, 30 µm. (**I**) Western blotting of BAG3, CRYAB, ANKRD1, and DES in ventricular extracts from 1-month-old *Hgs*-cKO and *Hgs*^fl/fl^ hearts. Quantification is shown in the lower panel. *p < 0.05, **p < 0.01 (means ± SEM, n = 4).

As defective autophagy/lysosome pathway would result in disruption of the proteolytic process and abnormal protein aggregation, we performed transmission electron microscopic analysis, and observed electron-dense perinuclear protein aggregates in *Hgs*-cKO myocardium (Figure 5A). Immunostaining for SEC61α, a marker of protein aggregation 29, also confirmed that both the abundance and size of protein aggregates were increased by *Hgs* knockout (Figure 5B). We also found that the autophagic substrate, SQSTM1, was aggregated in *Hgs* mutant hearts (Figure 5C). SQSTM1 can simultaneously bind to LC3 and ubiquitin, thereby linking ubiquitinated proteins to autophagosomes 30. Ubiquitinated proteins accumulation is one of the hallmarks of defective autophagy flux, and is also observed in LSDs. Fluorescent staining of ubiquitin showed increased ubiquitinated proteins in cardiomyocytes of *Hgs*-cKO mice compared with *Hgs*^fl/fl^ mice (Figure 5D). The upregulation of SQSTM1 and ubiquitinated proteins were also confirmed by western blot analysis (Figure 5E). Proteins linked to ubiquitin through K63 (K63-pUb) are found to be sorted and targeted to the ESCRT-mediated multivesicular body (MVB) pathway 31, but the upregulation of K63-pUb was far less than the upregulation of global ubiquitinated proteins in *Hgs*-cKO mice (Supplemental Figure 5), suggesting that deficiency of autophagy/lysosome pathway might be the main cause for the proteostasis impairment.

Proteotoxicity occurs when protein misfolding or aggregation impairs cellular function and can contribute to cardiomyopathy and heart failure 4. We constructed a gene set including 25 proteotoxicity-associated genes annotated in the OMIM and EntrezGene databases 5 and performed GSEA of proteomic data. The data revealed significant enrichment and upregulation of the proteotoxicity-associated pathway (Figure 5F). Immunofluorescence analysis confirmed that many of the proteins encoded by proteotoxicity-associated genes, including BAG3, CRYAB, and FLNC, were aggregated in *Hgs*-cKO hearts (Figure 5G-I). Western blot analysis also confirmed the upregulation of BAG3, CRYAB, ANKRD1, and DES in the *Hgs*-cKO hearts (Figure 5J).

Collectively, these findings indicated that *Hgs* deletion impaired proteostasis in cardiomyocytes.

### Inducible deletion of Hgs in adulthood led to RCM-like cardiomyopathy

To test whether HGS is also required for cardiac homeostasis in adulthood, we generated temporally controlled cardiomyocyte-specific *Hgs*-knockout mice by breeding *Hgs*^fl/fl^ mice with α*-*MHC-MerCreMer (hereafter referred to as MCM) transgenic mice that expressed a Cre recombinase in a tamoxifen-inducible and cardiomyocyte-specific manner 21. In MCM;*Hgs*^fl/fl^ mice treated with tamoxifen, we observed a significant reduction in *Hgs* mRNA and HGS protein levels in whole heart homogenates, compared with MCM;*Hgs*^+/+^ mice treated with tamoxifen (Supplemental Figure 6A-B). Cardiac fibrosis was observed in 3-month-old MCM;*Hgs*^fl/fl^ mice (Supplemental Figure 6C), and upregulation of fibrotic genes were also evident in the hearts of MCM;*Hgs*^fl/fl^ mice (Supplemental Figure 6D). The MCM;*Hgs*^fl/fl^ mice demonstrated increased cardiomyocyte apoptosis (Supplemental Figure 6E) and reactivation of the fetal genes encoding *Myh7*, *Nppa*, and *Nppb* (Supplemental Figure 6F). Echocardiographic analysis of tamoxifen-treated MCM;*Hgs*^fl/fl^ mice demonstrated a significant increase in left atrium internal diameter, a significant increase in the E/A ratio (≥ 2), and a significant decrease in EDT, suggesting abnormal LV relaxation and elevated filling pressure (Supplemental Figure 6G-J). Deletion of *Hgs* in adult cardiomyocytes also impaired the autophagy/lysosome pathway and caused aberrant aggregation of ubiquitinated proteins (Supplemental Figure 6K-L). However, we did not observe obvious calcification in MCM;*Hgs*^fl/fl^ mice, which might be due to the relative insufficient knockout efficiency by α-MHC-MCM compared with α-MHC-*Cre*. Taken together, these data demonstrated that postnatal deletion of cardiomyocyte *Hgs* also led to lysosome dysfunction and induced RCM-like cardiomyopathy.

### Hgs ablation resulted in RCM partially by the aberrant aggregation of proteins encoded by proteotoxicity-associated genes

**Figure 6:**
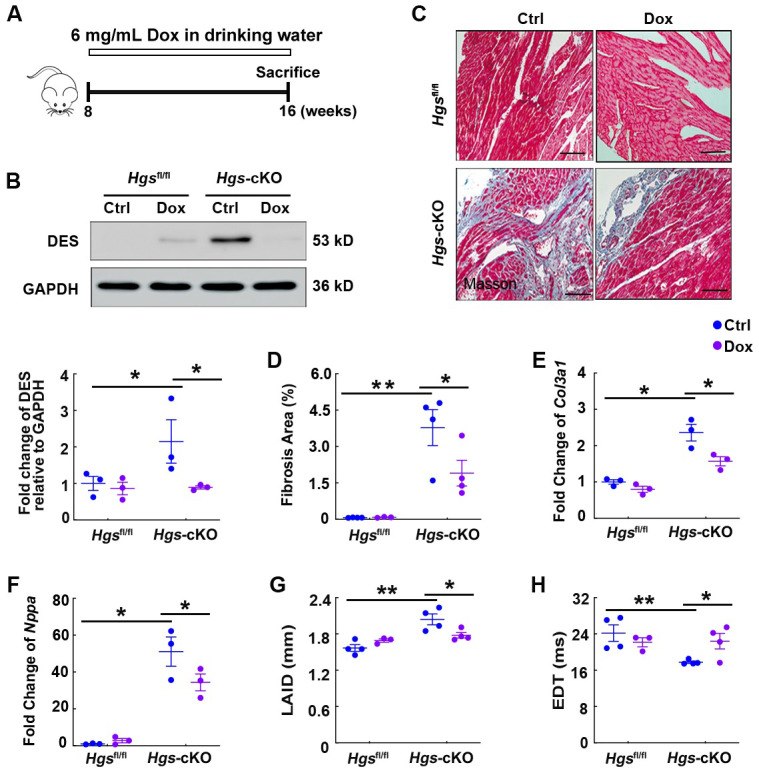
***Hgs* ablation resulted in RCM partially by the aberrant aggregation of proteins encoded by proteotoxicity-associated genes. (A)** Schematic representation of the rescue experiment. 8-week-old *Hgs*^fl/fl^ and *Hgs*-cKO mice were administrated with 6 mg/mL Dox for 8 weeks. Echocardiographic analyses were performed, and the animals were then sacrificed for histological and molecular analyses. **(B)** Western blotting of DES in hearts of *Hgs*^fl/fl^ and* Hgs*-cKO mice treated with Dox compared with that treated with sucrose (Ctrl). Quantification is shown below. *p < 0.05 (means ± SEM, n = 3). **(C)** Masson's trichrome staining of cardiac sections. Scale bars, 100 µm. **(D)** Quantification of fibrotic area. *p < 0.05, **p < 0.01 (means ± SEM, n = 4 for *Hgs*^fl/fl^ Ctrl, *Hgs*-cKO Ctrl and *Hgs*-cKO Dox groups; n = 3 for *Hgs*^fl/fl^ Dox group). **(E, F)** Real-time PCR analysis of *Col3a1* and* Nppa* mRNA levels in hearts. *p < 0.05 (means ± SEM, n = 3). **(G, H)** Measurements of the left atrium internal diameter (LAID) and E-wave deceleration time (EDT). **p < 0.01, *p < 0.05 (means ± SEM, n = 4 for *Hgs*^fl/fl^ Ctrl, *Hgs*-cKO Ctrl and *Hgs*-cKO Dox groups; n = 3 for *Hgs*^fl/fl^ Dox group).

Previous studies have revealed that accumulation of these proteins is harmful to the heart and that mutations in the genes encoding these proteins are associated with familial RCM 32-35. To prove our hypothesis that aberrant protein aggregations may cause RCM in *Hgs*-knockout mice, we used Dox, a well-established protein aggregation inhibitor 36, to treat 8-week-old* Hgs*^fl/fl^ and* Hgs*-knockout mice for 8 weeks (Figure 6A). Western blot analyses showed that Dox treatment reduced DES expression in *Hgs*-cKO hearts (Figure 6B). Furthermore, Dox treatment attenuated cardiac fibrosis caused by *Hgs* deletion, as revealed by Masson's trichrome staining, quantification of fibrotic areas, and detection of *Col3a1* mRNA expression (Figure 6, C-E). WGA staining of heart cross-sections revealed no significant change in cardiomyocyte size among different groups of mice (Supplemental Figure 7). Reactivation of the fetal genes encoding *Nppa* was also attenuated in *Hgs*-cKO Dox mice (Figure 6F). Echocardiography analyses revealed that the *Hgs*-cKO Ctrl group showed gradually disruption of diastolic dysfunction, whereas *Hgs*-cKO Dox mice showed partial recovery of diastolic dysfunction, as demonstrated by decreased left atrium internal diameter (LAID), and increased EDT (Figure 6, G-H and Supplemental Table 5). Collectively, these results suggested that reducing aberrant protein aggregation in cardiomyocytes attenuated the RCM-like cardiomyopathy of *Hgs*-knockout mice.

## Discussion

In this study, we demonstrated that loss of HGS in cardiomyocytes led to RCM-like cardiomyopathy in mice, indicating that HGS may be a causative gene for RCM. RCM has the poorest prognosis among all types of cardiomyopathies, and no effective treatments for RCM have been developed 37. A limited number of genes, including troponins, MYBP-C, MYH7, MYL2, MYL3, DES, and MYPN, are associated with RCM 38. In addition, only a few of mouse models of RCM have been established. Transgenic mice harboring the cardiac troponin I (cTnI) K178E and R192H mutations were found to show symptoms consistent with RCM in human patients carrying these mutations 39. Another study showed that RCM is caused by myofibril hypersensitivity to Ca^2+^ in cTnI mutant cardiomyocytes 40. The E143K mutation in the myosin essential light chain gene is associated with human RCM. Transgenic E143K mutation-bearing mice exhibit RCM phenotypes resulting from E143K-induced myosin hypercontractility 41. Mice with CryAB^R120G^-based proteinopathy recapitulate well the RCM phenotype (HFpEF) of human desmin-related cardiomyopathy 42. In the current study, we found that *Hgs* deletion in cardiomyocytes resulted in cardiac pathological changes that mirrored the pathologic hallmarks of RCM in humans. Our findings provided the first *in vivo* evidence of the causative link between *Hgs* deficiency and RCM.

LSDs are a group of inherited diseases caused by defects in lysosomal function 43. Heart is one of the major organs affected in LSDs, and many forms of LSD are accompanied by cardiac hypertrophy 43. Although several case reports have implicated the clinical association of LSD and RCM 8, 9, the experimental evidence and related mechanisms of this association are largely unknown. In this study, we found that *Hgs*-cKO mice displayed characteristic features of LSD: cholesterol and autophagosome accumulation, and lysosome enlargement. HGS has been identified as a regulator of intracellular cholesterol transporter in Hela cells 18. Our study confirmed this finding *in vivo* and further demonstrated that deficiency of *Hgs* caused lysosomal cholesterol storage disorder, suggesting the important role of *Hgs* deficiency in LSD-associated RCM.

We identified HGS as a critical regulator of autophagy in cardiomyocytes by regulating lysosomal homeostasis. Previous studies have revealed that several ESCRT proteins involve in autophagy/lysosome pathways. Knockdown of multivesicular body protein 2B (CHMP2B), a component of ESCRT-III, can restore autophagy and decrease proteotoxicity, thereby preventing cell death in atrogin-1-knockout mice 44. ESCRT-0 subunit HGS has been shown to regulate autophagy in HeLa cells and neurons 45, while the underlying mechanism remains unknown. Here, we showed that loss of *Hgs* caused impaired lysosomal homeostasis in cardiomyocytes. On the one hand, *Hgs* deficient hearts exhibited significant lysosomal damages characterized by increased number and size of lysosomes, as well as increased LGALS3/Galectin-3 puncta 25. On the other hand, accumulation of cholesterol was occurred in cardiomyocytes of *Hgs*-cKO mice. Cholesterol accumulation is associated with lysosomal dysfunction, and affects the fusion of lysosomes with endosomes and with autophagic vacuoles 26, 46. Consistently, impairment of autophagic flux was observed in *Hgs*-cKO hearts. We inferred that *Hgs* deficiency-caused defective endosomal cholesterol trafficking resulted in lysosomal dysfunction, which accounts for the impaired autophagic flux in the *Hgs*-cKO hearts.

As part of PQC system, autophagy/lysosome pathway maintains cellular proteostasis. Impaired proteostasis will cause aberrant protein aggregates and therefore proteotoxicity, which has been shown to be pathogenic in a large subset of cardiac diseases 4, 47. Here, we demonstrated that Dox treatment reduced the proteotoxicity-associated proteins, and restored diastolic function of *Hgs*-cKO mice. Thus, our data provided compelling evidence that aberrant protein aggregates caused RCM in *Hgs*-cKO mice, suggesting that clearance of protein aggregates may be a potential therapeutic strategy for RCM.

Our study had some limitations. Firstly, although our findings indicated that HGS regulated cardiac proteostasis and prevented RCM, the clinical relevance of HGS expression in RCM could not be established, since we were unable to obtain human RCM samples. Secondly, we only detected the significant attenuation of DES in *Hgs*-cKO mice under the treatment of Dox, suggesting that DES might mainly mediate the effect *Hgs* deletion on RCM. Further studies are required for treating RCM via developing more general proteostasis drugs.

In summary, this study revealed the essential role of HGS in cardiac homeostasis by modulating lysosome-mediated degradation of proteins, uncovering a possible causal link between defective HGS-mediated proteostasis and RCM. The cardiomyocyte-specific *Hgs*-knockout mouse model presented in this study could serve as an animal model for elucidating the mechanisms of LSD-associated RCM and developing target-oriented medications in the future.

## Supplementary Material

Supplementary figures and tables.
