# Supplementary material for: Hgs Deficiency Caused Restrictive Cardiomyopathy via Disrupting Proteostasis
Source: Int J Biol Sci. 2022 Feb 28;18(5):2018–31. doi: 10.7150/ijbs.69024 (PMC8935245; doi:10.7150/ijbs.69024)
Supplement: Supplementary file 1 — Supplementary figures and tables. [file ijbsv18p2018s1.pdf]

Supplementary Materials

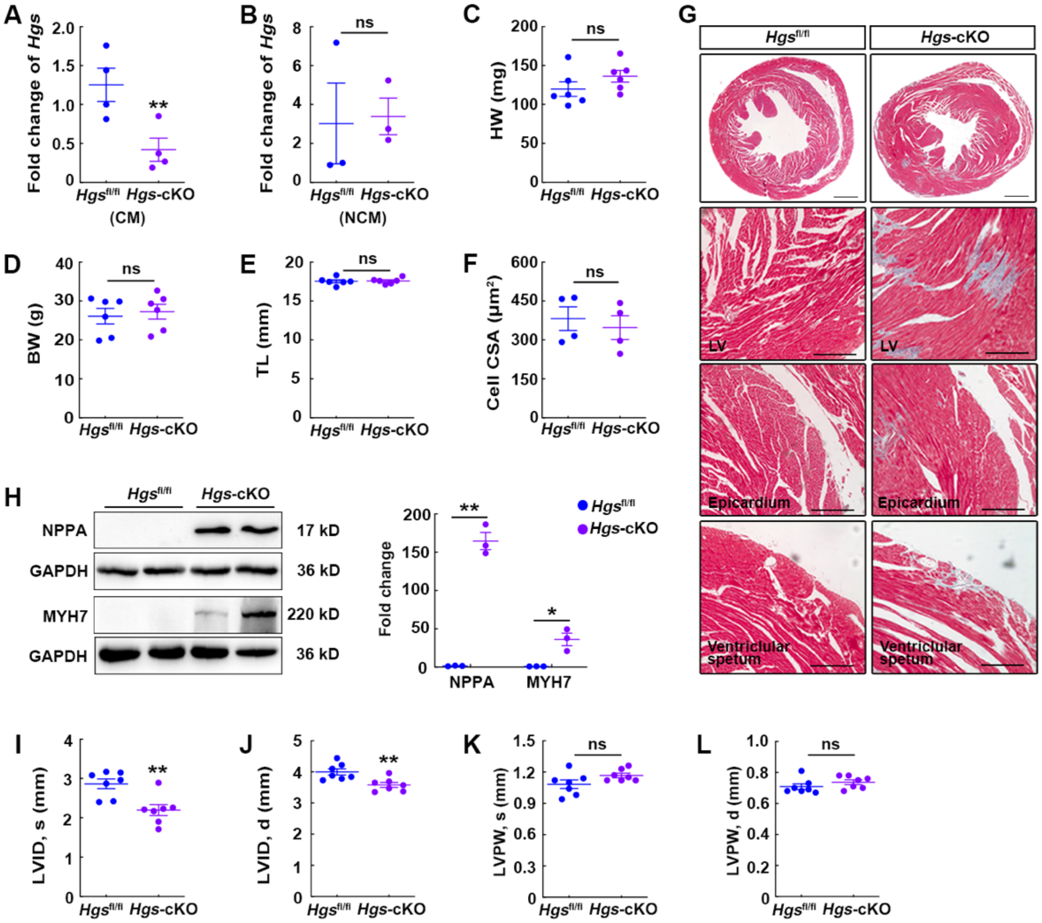

Supplementary Figure 1. Phenotypic analyses of 3-month-old *Hgs*$^{\mathbf{fl/fl}}$ and *Hgs*-cKO mice. (A) QPCR analysis of Hgs mRNA in adult cardiomyocytes from *Hgs*$^{fl/fl}$ and *Hgs*-cKO mice. $^{**}p < 0.01$ (means ± SEM, n = 4). **(B)** QPCR analysis of Hgs mRNA in adult non-cardiomyocytes from *Hgs*$^{fl/fl}$ and *Hgs*-cKO mice. (means ± SEM, n = 3). **(C)**Measurements of the HW in *Hgs*$^{fl/fl}$ and *Hgs*-cKO mice. **(D)** Measurements of the BW in *Hgs*$^{fl/fl}$ and *Hgs*-cKO mice. **(E)** Measurements of the TL in *Hgs*$^{fl/fl}$ and *Hgs*-cKO mice, (means ± SEM, n = 6). **(F)** Quantification of mean cardiomyocyte cross-section area (CSA) of ventricular cardiomyocytes. (means ± SEM, n = 4). **(G)** Masson's trichrome staining of sections from *Hgs*$^{fl/fl}$ and *Hgs*-cKO mice. Scale bars, column 1: 1.5 mm; columns 2-4: 100 μm. **(H)** Western blot analysis of NPPA and MYH7 expression in the extract of heart samples from *Hgs*$^{fl/fl}$ and *Hgs*-cKO mice. The quantification of the bands is shown on the

right. $^{**}p < 0.01$, $^{*}p < 0.05$ (means ± SEM, n = 4). **(I, J)** Measurements of the LV internal diameters in systole (LVID, s) and diastole (LVID, d). $^{**}p < 0.01$ (means ± SEM, n = 7). **(K, L)** Measurements of the LV posterior wall thickness in systole (LVPW, s) and diastole (LVPW, d), (means ± SEM, n = 7).

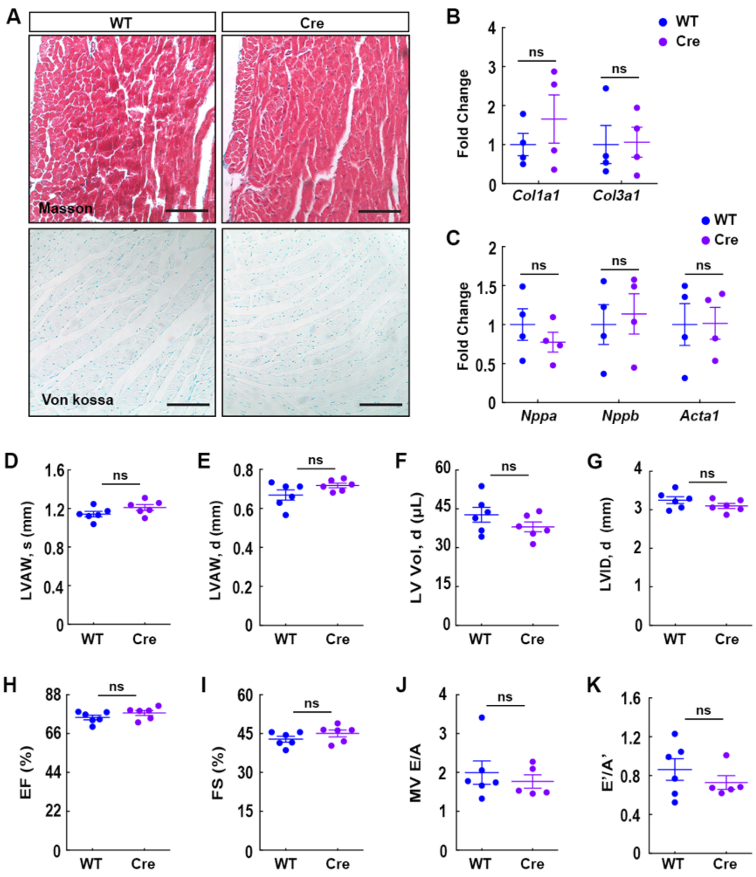

Supplementary Figure 2. There were no apparent differences between wild type (WT) and α-MHC-Cre transgenic (Cre) mice. (A) Masson's trichrome and Von kossa staining of sections from 3-month-old WT and Cre mice. Scale bars, 100 μm. **(B)** Real-time PCR analyses of *Col1a1*, and *Col3a1* mRNA levels in mice. (means ± SEM, n = 4). **(C)** Real-time PCR analyses of *Nppa*, *Nppb* and *Acta1* mRNA levels in mice. (means ± SEM, n = 4). **(D-I)** Measurements of LVAW in systole, LVAW in diastole, LVVol in diastole, LVID in diastole, EF, FS, MV E/A and E'/A' in 3-month-old WT and Cre mice. (means ± SEM, n = 6) **(J, K)** Measurements of MV E/A and E'/A' in 3-month-old WT and Cre mice. (means ± SEM, WT: n = 6, Cre: n = 5).

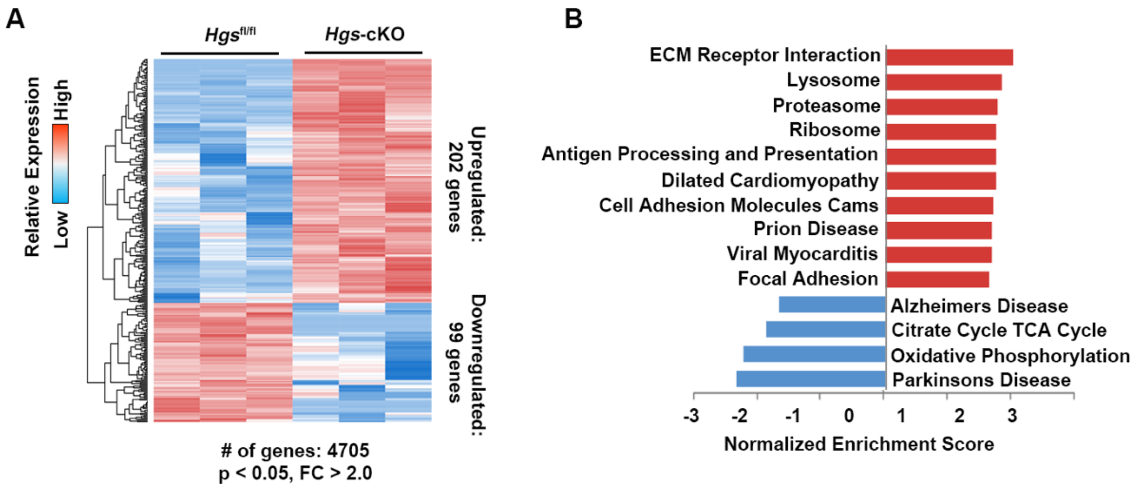

Supplementary Figure 3. Quantitative proteomic analysis of heart tissues from *Hgs*$^{fl/fl}$ and *Hgs*-cKO mice. (A) Gene clustering by Z-score using Euclidean distance metric in *Hgs*$^{fl/fl}$ and *Hgs*-cKO samples. Gene expression fold change (FC) was calculated for *Hgs*-cKO compared with the *Hgs*$^{fl/fl}$ mice. $p < 0.05$, FC > 2.0. **(B)** Enrichment plot showing the lysosome pathway enriched in *Hgs*-cKO mice.

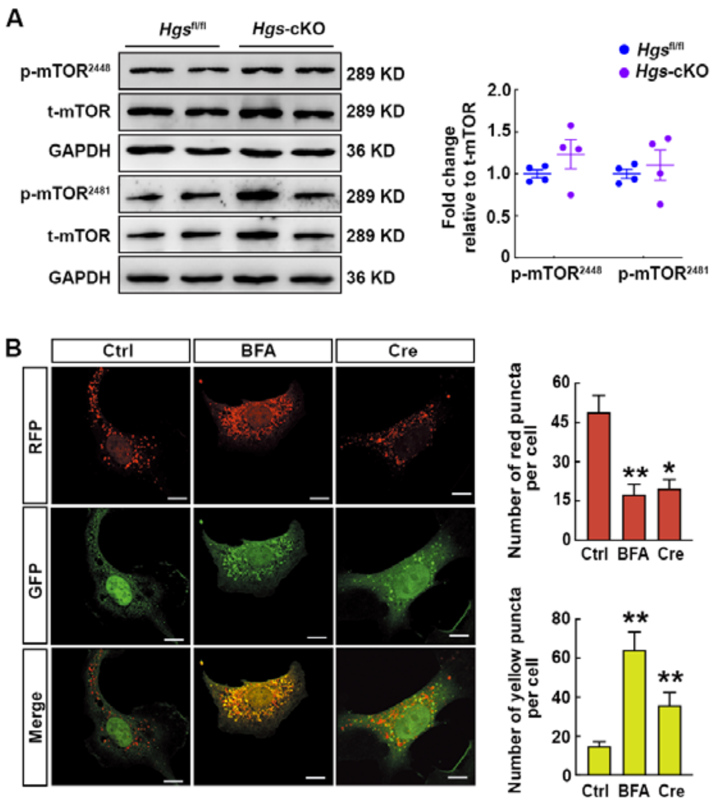

Supplementary Figure 4. *Hgs* deletion in cardiomyocytes impaired autophagy flux. (A) Western blotting of p-mTOR^{2448} and p-mTOR^{2481} expression in *Hgs*-cKO and *Hgs*$^{fl/fl}$ hearts. Quantification is shown on the right. **(B)** *Hgs*-knockout primary neonatal cardiomyocytes (Cre) infected with adenovirus encoding RFP-GFP-LC3B showed blocked fusion of autophagosomes with lysosomes, whereas the control cardiomyocytes (Ctrl) showed a basal level of autophagosomes and autolysosome formation. Bafilomycin A1 (BFA)-treated cells served as positive controls. Scale bars, 10 μm. The quantifications of RFP single positive and RFP$^+$GFP$^+$ puncta in *Hgs*-knockout cells and control cells. Puncta from 27 cells per group were scored and analyzed by microscopy for their fluorescence color. *$p < 0.05$, **$p < 0.01$.

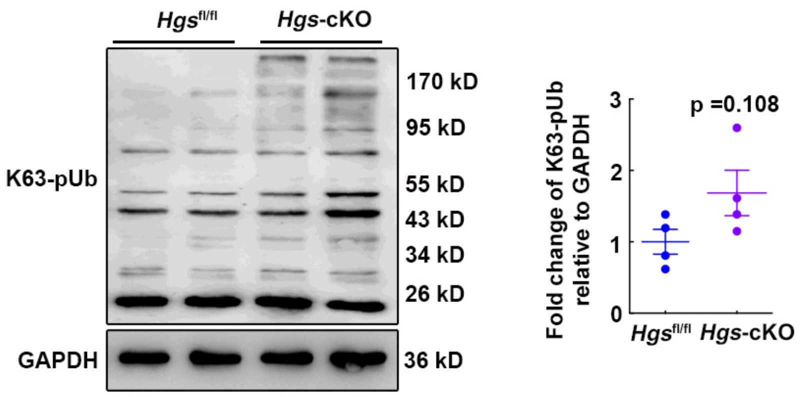

Supplementary Figure 5. *Hgs* deletion in cardiomyocytes did not cause the upregulation of K63-linked polyubiquitinated proteins. Western blotting of K63-linked polyubiquitinated proteins in *Hgs*-cKO and *Hgs*$^{fl/fl}$ hearts. Quantification is shown right.

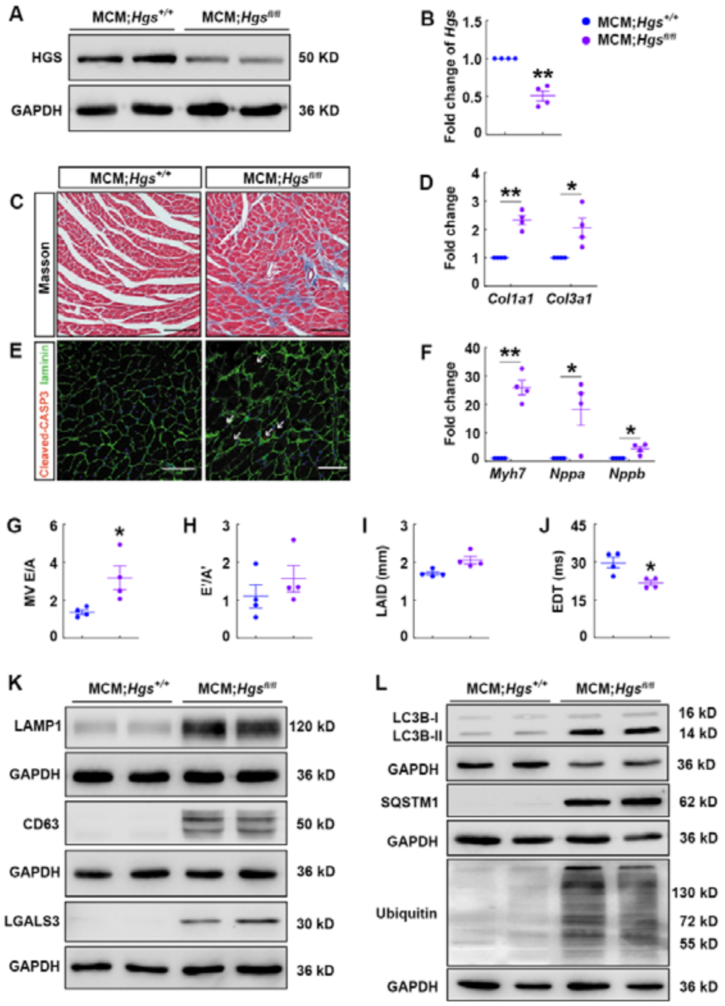

Supplementary Figure 6. Inducible deletion of Hgs in adulthood induced RCM-like cardiomyopathy. (A) Western blotting of HGS expression in MCM; $Hgs^{+/+}$ and MCM;$Hgs^{fl/fl}$ hearts. **(B)** Real-time PCR analysis of Hgs mRNA levels in MCM; $Hgs^{+/+}$ and MCM;$Hgs^{fl/fl}$ mice. **p < 0.01 (means ± SEM, n = 4). **(C)** Masson's trichrome staining of sections from MCM;$Hgs^{+/+}$ and MCM;$Hgs^{fl/fl}$ hearts. Scale bars, 100 μm. **(D)** Real-time PCR analysis of Col1a1 and Col3a1 mRNA levels in MCM;$Hgs^{+/+}$ and MCM;$Hgs^{fl/fl}$ mice. *p < 0.05, **p < 0.01 (means ± SEM, n = 4). **(E)** Immunofluorescence analysis of ventricular sections costained with anti-laminin antibodies (green) and antibodies against cleaved-CASP3 (red). Scale bars, 100 μm. **(F)** Real-time PCR analysis of Myh7, Nppa and Nppb mRNA levels in MCM; $Hgs^{+/+}$ and MCM;$Hgs^{fl/fl}$ mice. *p < 0.05, **p < 0.01 (means ±

SEM, n = 4). **(G-J)** Measurements of mitral valve E/A, E'/A', LAID and EDT of MCM; $Hgs^{+/+}$ and MCM;$Hgs^{fl/fl}$ mice. *$p < 0.05$ (means ± SEM, n = 4). **(K)** Western blotting of LAMP1, CD63 and LGALS3 in ventricular extracts from MCM; $Hgs^{+/+}$ and MCM;$Hgs^{fl/fl}$ mice. **(L)** Western blotting of LC3B, SQSTM1and ubiquitinated protein in ventricular extracts from MCM; $Hgs^{+/+}$ and MCM;$Hgs^{fl/fl}$ mice.

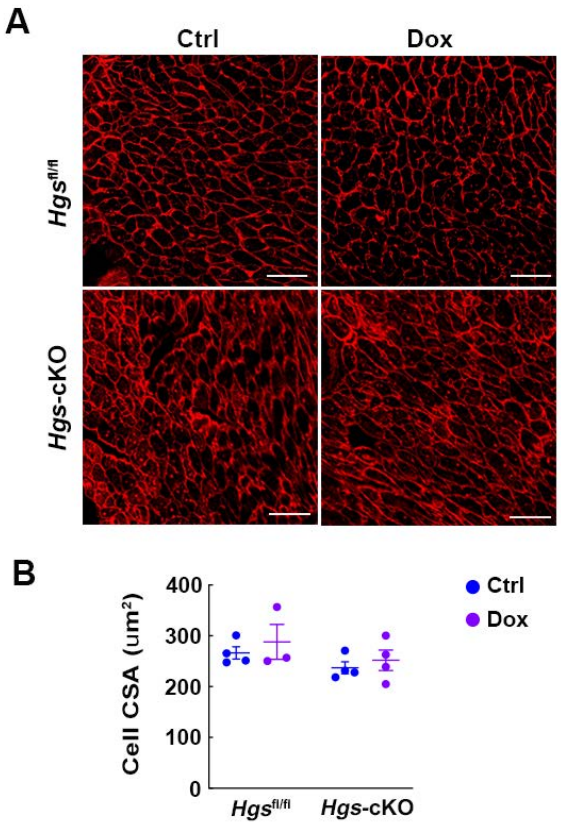

Supplementary Figure 7. Cell size was not affected by Dox treatment. (A) WGA staining was performed on transverse sections of myocardium from the *Hgs*$^{fl/fl}$ and *Hgs*-cKO mice treated with sucrose (Ctrl) or Dox. Scale bars, 40 μm. **(B)** Quantification of mean cardiomyocyte cross-section area (CSA) of ventricular cardiomyocytes, (means ± SEM, n = 4 for *Hgs*$^{fl/fl}$ Ctrl, *Hgs*-cKO Ctrl and *Hgs*-cKO Dox groups; n = 3 for *Hgs*$^{fl/fl}$ Dox group).

Supplementary Table 1. Summary of birth rate and death rate in mice

	genotype				
	Hgs$^{fl/+}$	*Hgs*$^{fl/fl}$	*α-MHC-Cre;Hgs*$^{fl/+}$	*α-MHC-Cre;Hgs*$^{fl/fl}$	Total
Birth rate	38/176 (21.6%)	36/176 (20.5%)	48/176 (27.3%)	54/176 (30.7%)	176
Death rate		0/20		0/20	40

Supplementary Table 2. M-mode echocardiographic characterization of *Hgs*$^{fl/fl}$ and *Hgs*-cKO mice

	Hgs$^{fl/fl}$ (n = 7)	*Hgs*-cKO (n = 7)
LV volume in diastole, μL	70.28 ± 4.22	53.96 ± 2.95**
LV volume in systole, μL	31.82 ± 3.16	16.87 ± 2.78**
LV internal diameter in diastole, mm	4.00 ± 0.10	3.58 ± 0.08**
LV internal diameter in systole, mm	2.86 ± 0.12	2.20 ± 0.14**
LA internal diameter, mm	2.00 ± 0.11	2.58 ± 0.03**
Posterior wall thickness in diastole, mm	0.71 ± 0.02	0.74 ± 0.02
Posterior wall thickness in systole, mm	1.08 ± 0.04	1.17 ± 0.02
Anterior wall thickness in diastole, mm	0.70 ± 0.02	0.74 ± 0.02
Anterior wall thickness in systole, mm	1.07 ± 0.03	1.17 ± 0.03
Ejection fraction, %	54.79 ± 3.95	69.63 ± 3.22*
Fraction shortening, %	28.40 ± 2.54	38.90 ± 2.51*
LV mass, mg	80.04 ± 4.83	70.74 ± 3.56
Heart rate, bpm	412.71 ± 3.61	410.43 ± 3.94

The results presented are means ± SEM. *$p < 0.05$, **$p < 0.01$ vs *Hgs*$^{fl/fl}$ mice.

Supplementary Table 3. Doppler echocardiographic characterization of *Hgs*$^{fl/fl}$ and *Hgs*-cKO

	Hgs$^{fl/fl}$ (n = 7)	*Hgs*-cKO (n = 7)
Mitral inflow		
E, mm/s	669.97 ± 75.52	693.29 ± 47.87
A, mm/s	428.18 ± 50.01	143.21 ± 15.40 **
E/A	1.80 ± 0.19	4.99 ± 0.52 **
EDT, ms	32.87 ± 1.63	20.81 ± 1.16 **
Septal mitral annulus		
E’, mm/s	23.50 ± 3.52	26.88 ± 3.45
A’, mm/s	21.51 ± 3.01	9.34 ± 0.69 **
E’/A’	1.24 ± 0.25	2.93 ± 0.33 **
Heart rate, bpm	326.86 ± 13.32	285.14 ± 6.66

The results presented are means ± SEM. **$p < 0.01$ vs *Hgs*$^{fl/fl}$ mice.

Supplementary Table 4. KEGG pathways enriched in *Hgs*-cKO vs *Hgs*$^{fl/fl}$ mice by GSEA analysis

Upregulated					
Rank	KEGG pathways	Size	NES	FDR	Top genes in core enrichment
1	ECM receptor interaction	33	2.00	0.006	Col6a2, Itga5, Dag1, Col1a2, Col6a1, Lamc1, Col6a3, Itgb1, Lamb1, Lama2
2	Lysosome	46	1.83	0.031	Ctsd, cd63, Gaa, Lgmn, Clta, Lamp1, Hexb, Psap, Laptm4a, Gns
3	Proteasome	39	1.75	0.068	Psmd8, Psmd3, Psmc3, Psmc4, Psmd13, Psmb3, Psmd6, Psmc1, Psmd12, Psma4
4	Ribosome	64	1.74	0.062	Rpl13, Rpl12, Rpl35a, Rpl3, Rps12, Rps7, Rps13, Rps25, Rpsa, Rps8
5	Antigen processing and presentation	18	1.73	0.055	Creb1, Lgmn, Hsp90aa1, Hsp90ab1, Pdia3, Hspa8, Calr, Hspa1b, Ctsb
6	Dilated cardiomyopathy	39	1.73	0.050	Itga5, Dag1, Actg1, Itgb1, Lama2, Myh7, Itgav, Des, Lmna, Sgcd
7	Cell adhesion molecules	18	1.69	0.063	Jam2, Itgb1, Ncam1, Itgav, Ptprm, Neo1, Itga9, Itgb2, Cdh5, F11r
8	Prion diseases	18	1.66	0.075	Prnp, Lamc1, C1qc, Ncam1, Mapk3, Map2k1, Map2k2, Stip1
9	Viral myocarditis	27	1.66	0.069	Dag1, Actg1, Cxadr, Lama2, Myh7, Sgcd, Sgcb, Sgca, Itgb2, Myh3
10	Focal adhesion	81	1.63	0.085	Col6a2, Itga5, Actg1, Col1a2, Col6a1, Lamc1, Col6a3, Itgb1, Capn2, Mapk3
11	Starch and sucrose metabolism	16	1.61	0.088	Gbe1, Gaa, Ugp2, Enpp1, Pgm1, Pygb
12	Hypertrophic cardiomyopathy	40	1.61	0.084	Itga5, Dag1, Actg1, Itgb1, Lama2, Myh7, Itgav, Des, Lmna, Sgcd
13	Prostate cancer	24	1.58	0.106	Creb1, Mapk3, Hsp90aa1, Hsp90ab1, Hsp90b1, Map2k1, Map2k2, Pdgfrb, Ikbkg
14	Glutathione metabolism	30	1.53	0.152	Gstm5, Gstm1, Pgd, Gsr, Gclm
15	Regulation of actin cytoskeleton	83	1.51	0.165	Itga5, Actg1, Slc9a1, Chrm2, Itgb1, Cyfip1, Mapk3, Myl12b, Arpc1b, Itgav
16	Melanogenesis	22	1.50	0.164	Mapk3, Gnao1, Map2k1, Map2k2, Gnaq, Adcy5, Gnai2
17	Fc gamma R-mediated phagocytosis	34	1.50	0.159	Plcg2, Marcksl1, Dnm1, Mapk3, Arpc1b, Cfl1, Arpc5, Cfl2, Gsn, Rps6kb2
18	Axon guidance	30	1.49	0.158	Plxna1, Plxnb2, Itgb1, Mapk3, Cfl1, Cfl2, Ntn1, Epha4
19	Small cell lung cancer	23	1.46	0.202	Lamc1, Itgb1, Lamb1, Lama2,

					Itgav, Col4a2, Lama4, Lamb2, Cdk6, Ikbkg
Downregulated					
Rank	KEGG pathways	Size	NES	FDR	Top genes in core enrichment
1	Parkinsons disease	79	-2.34	0.002	Nd2, Ndufs4, Ndufa2, Sdha, Sdhb, Nd1, Cox1, Ndufs6, Ndufab1, Uqcrb
2	Oxidative phosphorylation	81	-2.24	0.002	Nd2, Ndufs4, Ndufa2, Sdha, Sdhb, Nd1, Cox1, atp6v1b2, Ndufs6, Ndufab1
3	Citrate cycle TCA cycle	28	-1.87	0.020	Suclg1, Sdha, Sdhb, Idh3g, Mdh2, Cs, Sucla2, Suclg2, Fh, Aco1
4	Alzheimers disease	88	-1.68	0.077	Ndufs4, Ndufa2, Sdha, Sdhb, Cox1, Ndufs6, Ndufab1, Bad, Uqcrb, Ndufa5

NES, normalized enrichment score; FDR, false discovery rate. FDR < 0.25 was considered as significantly enriched.

Supplementary Table 5. Echocardiographic characterization of mice treated with Dox

	0 day				60 days			
	Hgs$^{fl/fl}$ **Ctrl**	***Hgs***$^{fl/fl}$ **Dox**	***Hgs*-cKO Ctrl**	***Hgs*-cKO Dox**	***Hgs***$^{fl/fl}$ **Ctrl**	***Hgs***$^{fl/fl}$ **Dox**	***Hgs*-cKO Ctrl**	***Hgs*-cKO Dox**
	**(n = 4)**	**(n = 3)**	**(n = 4)**	**(n = 4)**	**(n = 4)**	**(n = 3)**	**(n = 4)**	**(n = 4)**
**LV volume in diastole, μL**	48.95 ± 2.48	55.88 ± 2.28	46.81 ± 4.99	54.44 ± 3.47	46.23 ± 2.43	42.27 ± 2.96	51.98 ± 3.78	48.70 ± 5.52
**LV volume in systole, μL**	14.09 ± 0.69	16.95 ± 2.68	11.51 ± 2.21	13.12 ± 1.11	11.86 ± 1.14	9.50 ± 0.31	11.00 ± 2.07	9.11 ± 2.04
**LVID in diastole, mm**	3.44 ± 0.07	3.64 ± 0.06	3.36 ± 0.16	3.59 ± 0.09	3.36 ± 0.07	3.23 ± 0.10	3.52 ± 0.10	3.42 ± 0.16
**LVID in systole, mm**	2.08 ± 0.04	2.22 ± 0.14	1.88 ± 0.16	2.02 ± 0.07	1.94 ± 0.08	1.78 ± 0.02	1.86 ± 0.14	1.71 ± 0.16
**LAID, mm**	1.57 ± 0.02	1.54 ± 0.01	2.02 ± 0.05**	2.09 ± 0.17*	1.57 ± 0.04	1.69 ± 0.02	2.04 ± 0.07**	1.78 ± 0.04*$^{\#}$
**Ejection fraction, %**	71.20 ± 0.20	70.08 ± 3.42	72.99 ± 3.00	75.89 ± 1.60*	74.23 ± 2.60	77.23 ± 1.59	77.87 ± 2.64	82.25 ± 2.21
**Fraction shortening, %**	39.55 ± 0.17	39.05 ± 2.66	44.36 ± 2.67	43.87 ± 1.48*	42.34 ± 2.09	44.77 ± 1.57	47.49 ± 2.64	50.28 ± 2.31
**Heart rate, bpm**	419 ± 5.68	413.33 ± 5.89	409.75 ± 7.73	401.5 ± 5.34	431 ± 14.84	430.67 ± 9.62	419.5 ± 6.62	426.0 ± 16.66
**MV E/A**	1.75 ± 0.32	1.60 ± 0.17	3.51 ± 0.44**	9.29 ± 2.86**	1.48 ± 0.04	1.94 ± 0.21	7.68 ± 3.42	5.22 ± 1.54
**EDT, ms**	25.70 ± 1.18	22.88 ± 2.07	18.44 ± 0.99*	17.56 ± 0.92**	23.04 ± 1.64	22.16 ± 0.82	17.76 ± 0.22*	22.40 ± 1.49$^{\#}$
**E'/A'**	0.86 ± 0.09	0.82 ± 0.15	2.39 ± 0.12*	2.82 ± 0.48*	0.88 ± 0.07	1.14 ± 0.17	2.31 ± 0.23*	2.06 ± 0.11
**Heart rate, bpm**	388.75 ± 8.78	346.33 ± 30.51	293.75 ± 26.33	284 ± 10.80*	395.33 ± 13.01	383.33 ± 16.66	357.75 ± 17.68	321 ± 24.57

The results presented are means ± SEM. *p < 0.05, **p < 0.01 vs *Hgs*$^{fl/fl}$ Ctrl group. $^{\#}$p< 0.05, vs *Hgs*-cKO Ctrl group.
